# Familial gastric cancer: detection of a hereditary cause helps to understand its etiology

**DOI:** 10.1186/1897-4287-10-18

**Published:** 2012-12-12

**Authors:** Ingrid P Vogelaar, Rachel S van der Post, Tanya M Bisseling, J Han JM van Krieken, Marjolijn JL Ligtenberg, Nicoline Hoogerbrugge

**Affiliations:** 1 Department of Human Genetics, Radboud University Nijmegen Medical Centre, PO box 9101, Nijmegen, 6500HB, The Netherlands; 2 Department of Pathology, Radboud University Nijmegen Medical Centre, PO box 9101, Nijmegen, 6500HB, The Netherlands; 3 Department of Gastroenterology, Radboud University Nijmegen Medical Centre, PO box 9101, Nijmegen, 6500HB, The Netherlands

**Keywords:** Gastric cancer, Genetics, Hereditary diffuse gastric cancer, *CDH1*, E-cadherin

## Abstract

Worldwide, gastric cancer is one of the most common forms of cancer, with a high morbidity and mortality. Several environmental factors predispose to the development of gastric cancer, such as *Helicobacter pylori* infection, diet and smoking. Familial clustering of gastric cancer is seen in 10% of cases, and approximately 3% of gastric cancer cases arise in the setting of hereditary diffuse gastric cancer (HDGC). In families with HDGC, gastric cancer presents at relatively young age. Germline mutations in the *CDH1* gene are the major cause of HDGC and are identified in approximately 25-50% of families which fulfill strict criteria. Prophylactic gastrectomy is the only option to prevent gastric cancer in individuals with a *CDH1* mutation. However, in the majority of families with multiple cases of gastric cancer no germline genetic abnormality can be identified and therefore preventive measures are not available, except for general lifestyle advice. Future research should focus on identifying new genetic predisposing factors for all types of familial gastric cancer.

## General introduction

With an estimated 900,000 new cases per year (8.6% of all new cancer cases except skin cancer), gastric cancer (GC) is the fourth most common form of cancer worldwide. Even though the incidence is rapidly declining in the western world, it is still the second most common cause of death from cancer, with 740,000 deaths annually and a 5-year survival of 20% [1]. High-risk areas include East Asia (Japan, China and Korea), Eastern Europe, and parts of Central and South America. Incidence rates are low in North Europe, North America and Australia [1]. With a mean age at diagnosis above 60 years, gastric cancer is predominantly a disease of the elderly [2]. Only 6–7% of patients with gastric cancer present before the age of 50, and less than 2% before age 40 [2,3].

Gastric carcinoma is a heterogeneous disease, which is reflected by the diversity of the various histopathological classification schemes [4]. The most commonly used are those of the WHO [4] and Laurén [5]. The practical scheme of Laurén divides GC roughly into three main types; the diffuse type, the intestinal type and a rest group composed of mixed and indeterminate type [5]. Intestinal GC shows glandular or tubular components with various degrees of differentiation. Diffuse GC consists of poorly cohesive single cells without gland formation. Often signet ring cells are present; therefore it is also referred to as signet ring cell carcinoma [4]. In North America the distribution of the different subtypes is approximately 50% pure intestinal, 35% pure diffuse and 15% mixed diffuse-intestinal [6].

## Etiology of gastric cancer: environmental factors

Gastric cancer is a multifactorial disease, resulting from a combination of environmental factors and genetic alterations. Environmental factors are mainly involved in the etiology of the intestinal type of GC. The main environmental factor involved is *Helicobacter pylori* (*H.Pylori*) infection, which is commonly acquired during childhood and persists unless eradicated [7]. *H.Pylori* can induce a sequence of gastritis, intestinal metaplasia, dysplasia and eventually gastric cancer [8]. A meta-analysis of 12 studies revealed that infection with *H.pylori* increases the risk of developing GC about sixfold [9] and the WHO has classified *H.pylori* as a class I carcinogen in 1994 [10,11].

Cigarette smoking is an important behavioral risk factor for the development of GC. A large systematic review of 42 studies showed that the risk of GC is increased by 60% in male and 20% in female smokers compared to never smokers [12]. Smoking also enhances the carcinogenic effect of infection with *H.pylori*[13]. Another important risk factor for the development of GC is diet. An adequate intake of fruit and vegetables likely reduces the risk for developing GC [4]. Salt intake, on the other hand, is strongly associated with an enhanced risk to develop gastric carcinoma. Therefore, diet adjustments that reduce salt intake, for instance after replacement of salt preservation of food by refrigerators, are important factors in the decrease of the incidence of GC, [4]. Smoked meat and fish, pickled vegetables and chili peppers are also associated with GC in some populations [4]. Alcohol consumption has been studied in several populations, but results have been inconclusive [4].

The incidence of GC is declining worldwide, which is mainly due to the decline in the incidence of the distal, intestinal type of GC. The incidence of diffuse GC, for which no clear environmental risk factors are known, has not decreased [4]. In young people, in whom carcinomas are more likely to be due to genetic susceptibility, a greater proportion shows the diffuse type, suggesting that especially in this subtype germline genetics play a role [4].

## Etiology of gastric cancer: genetic factors

Familial aggregation of gastric cancer is known to occur in approximately 10% of the patients [14]. Epidemiologic studies have shown that in the general population the risk of gastric cancer in first-degree relatives with any type of gastric cancer is increased 2–3 fold [15]. As yet, however, in the vast majority of these patients the underlying genetic cause remains unknown. The most important GC susceptibility gene is *CDH1*, which accounts for 1-3% of gastric cancers [16]. Predisposing *CDH1* mutations have been encountered in about 30% of strictly selected Hereditary Diffuse Gastric Cancer (HDGC) families [17,18]. Moreover, *CDH1* germline mutations may also occur in approximately 7% of patients diagnosed before 50 years of age with tumors exhibiting either a diffuse or a mixed histology [19]. The recognition, surveillance and treatment of *CDH1* mutation carriers are extensively described below.

### Gastric cancer in familial intestinal gastric cancer and other hereditary cancer syndromes

Many families with intestinal type GC exhibiting an autosomal dominant inheritance pattern have been documented. However, in such families, disease causing germline mutations for intestinal GC have not been found yet.

An increased risk of developing both diffuse and intestinal type GC has been shown in several well known hereditary cancer syndromes, besides HDGC. These syndromes include Lynch syndrome [20-22], Peutz-Jeghers syndrome [23], Li-Fraumeni syndrome [24-26], hereditary breast and ovarian cancer [27,28], familial adenomatous polyposis (FAP) [29-31], MUTYH-associated adenomatous polyposis (MAP) [32], juvenile polyposis syndrome [33], and Cowden syndrome [34]. The lifetime risk of GC in these syndromes varies substantially between populations studied, but is generally low. For example, although benign gastric abnormalities such as fundic gland polyps develop in approximately 12.5-84% of FAP patients, only 40% of these polyps exhibit adenomatous features and an even smaller percentage (around 0.5%) develops into gastric adenocarcinoma [35]. In Lynch syndrome, the lifetime risk of GC varies between 2.1% in the Netherlands to 30% in Korea [36]. Clearly, the risk of developing GC in these syndromes is higher in areas with high incidence of GC in the general population, such as East Asia, indicating that in these types of hereditary forms of GC environmental factors may play a substantial role. Thus, in all these families life style advice is important, although its effect on GC risk is not precisely known. In most of the mentioned syndromes no consensus exists about recommendations of surveillance of the stomach.

### Identification of new genes underlying hereditary gastric cancer

In approximately two thirds of families fulfilling the strict HDGC criteria, no *CDH1* mutation is found and they remain genetically unexplained. Most of these families might carry mutations in other, still to be identified, GC susceptibility genes. As binding partner for E-cadherin, mutated β- and γ-catenin have been considered as candidates for diffuse GC predisposition [37]. The β-catenin gene (*CTNNB1*) was recently assessed in a series of 40 families with positive history of GC from the Netherlands without finding any mutations [Vogelaar et al., unpublished data, 2012].

Also in families with intestinal type GC exhibiting an autosomal dominant inheritance pattern, genetic susceptibility genes may play a role. No gene has been associated with this type of GC yet. In carefully selected patients next generation sequencing based techniques that allow for exome or even genome wide detection of genetic aberrations, might be exploited to unravel genetic predisposition in an unbiased way.

## Hereditary diffuse gastric cancer caused by germline *CDH1* mutations

In 1998, Guilford *et al*. identified germline mutations in the *CDH1* gene as a cause of hereditary diffuse gastric cancer (HDGC) [38]. *CDH1* encodes the protein E-cadherin, which plays an important role in cell–cell adhesion and the maintenance of epithelial integrity [39]. The mutation detection rate is approximately 50% in families with two gastric cancers in first-degree relatives with at least one diffuse gastric cancer (DGC) diagnosed before age 50, or three or more DGC in close relatives diagnosed at any age [18]. The percentage decreases if also single cases of DGC below the age of 35 are included [17]. Germline *CDH1* mutations are found in all ethnic groups [40]. The most common types of mutation are small insertions or deletions (35% of the mutations). Missense mutations occur in 28% of families, nonsense mutations and splice site mutations are both observed in 16% of families. Large exonic deletions are relatively rare, with a frequency of about 5% [41].

For both men and women, *CDH1* mutation carriers have a cumulative risk of gastric carcinoma by 80 years of age of 80%, with a mean age at diagnosis of 40 years. Additionally, women carrying a *CDH1* mutation have a 60% lifetime risk for developing lobular breast cancer [40].

### Genetic counseling and criteria for *CDH1* mutation testing

Genetic counseling is an essential component of the management of HDGC. It includes the analysis of the family history of at least three generations and histopathological confirmation of gastric (pre-) malignancies. The revised international criteria as established by the International Gastric Cancer Linkage Consortium (IGCLC) to select patients with an increased risk of familial gastric cancer for *CDH1* mutation testing are shown in Table 1[40]. Genetic testing is preferably initiated in an affected relative. In most countries the youngest age at which relatives at risk should be offered testing is set at age 18. Rare cases of gastric cancer before age 18 have been reported, but the overall risk of DGC before the age of 20 is very low [42,43].

**Table 1:** **Clinical criteria for testing for *CDH1 *germline mutations **[40]

•	1 diffuse gastric cancer case below age 40, or
•	2 gastric cancer cases in a family, one confirmed diffuse gastric cancer below age 50, or
•	3 confirmed diffuse gastric cancer cases in 1st or 2nd degree relatives independent of age, or
•	Personal or family history of diffuse gastric cancer and lobular breast cancer, with one diagnosis below age 50

### Proposed mechanism of HDGC initiation

In 2009, Humar and Guilford proposed a mechanism of HDGC initiation [44]. E-cadherin is known to play an important role in cell polarity and epithelial tissue architecture [45,46]. It is proposed that mutations in *CDH1* disturb the cell-cell adhesion mediated by E-cadherin, which causes disruption of the correct spatial organization of the cells. This in turn may interfere with processes that regulate cell division, such as the orientation of the mitotic spindle. Abrogated cell polarity may also lead to the disruption of cell fate determination [44,47,48]. These disturbed processes can ultimately result in the displacement of cells with self-renewal capacity into the lamina propria and lead to the formation of signet ring cell carcinomas with the capacity for sustained cell division and thus to progression [44].

### Prophylactic total gastrectomy in *CDH1* mutation carriers

Prophylactic gastrectomy is currently the only option to eliminate risk of GC development in *CDH1* mutation carriers [49]. The prognosis of patients with a prophylactic gastrectomy is very good. The estimated overall mortality for total gastrectomy is 2–4% with a nearly 100% risk of long-term morbidity. Associated problems following gastrectomy include abdominal pain after eating, dumping syndrome, lactose intolerance, fat malabsorption and steatorrhoea and postprandial fullness [40,50-52]. The optimal timing of prophylactic gastrectomy in individuals with *CDH1* mutations is not yet known. Preventive gastrectomy specimens of *CDH1* mutation carriers reveal multiple small signet ring cell lesions with low proliferation rates; few of these lesions progress to an aggressive carcinoma beyond the muscular mucosa [53]. It is unknown why only some of these lesions develop into aggressive carcinomas. No correlation between patient age and number of small signet ring cell foci has been observed. Blair *et al.* advise *CDH1* mutation carriers with normal gastric biopsies to consider gastrectomy once the individuals are older than 20 years of age [43]. Other authors recommend considering preventive gastrectomy when the *CDH1* mutation carrier is 5 years younger than the youngest family member with DGC, which generally means that preventive gastrectomy is postponed to an age later than 18 years [54].

In case of a preventive gastrectomy, total gastrectomy with Roux-en-Y reconstruction is recommended. There is no need for a radical lymph node dissection in the prophylactic setting since mucosal adenocarcinomas without submucosal invasion have a low risk of lymph node metastases [55].

### Pathological analysis of preventive gastrectomy specimens

Pathological analysis of the entire gastrectomy sample includes a thorough assessment microscopically with haematoxylin and eosin (H&E) and a mucin stain, such as periodic acid-Schiff (PAS). PAS-staining has proven to be helpful as a primary stain, increasing the detection rate of small invasive signet ring cell foci and reducing screening time [56]. The ‘Swiss roll’ technique can be used to include the complete mucosa [57]. The pathology report should mention all gastric abnormalities and localization, as (pre-) malignant lesions, intestinal metaplasia, dysplasia, inflammation and presence of *H.pylori*-associated gastritis. Histological confirmation of resection margins consisting of proximal esophageal and distal duodenal mucosa is essential, since new GC can develop in remaining gastric mucosa.

### Pathology of HDGC

**Figure 1:**
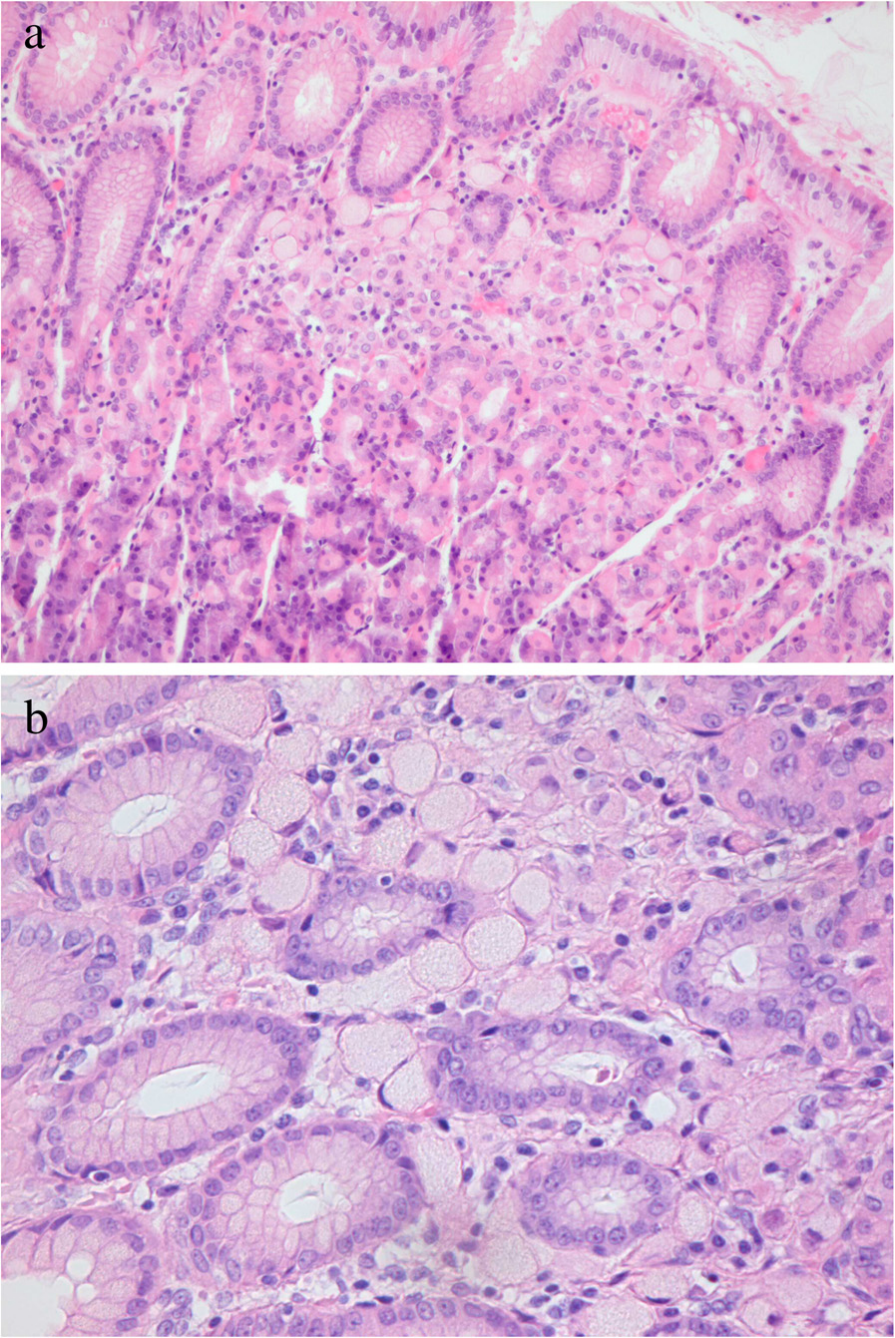
**Intramucosal signet ring cell carcinoma. a.** Typical small intramucosal focus of a signet ring cell carcinoma in a preventive gastrectomy specimen from a CDH1 mutation carrier (H&E, magnification 100x). **b.** Detail of signet ring cells between normal foveolar glands and a normal background without signs of gastritis. (H&E, magnification 200x).

Pathological mapping of complete gastrectomy specimens has shown that early-stage HDGC is characterized by the presence of a few to up to hundreds foci of stage T1a signet ring cell carcinoma (SRCC) restricted to the superficial lamina propria, without nodal metastases (Figure 1a and 1b) [43,54,58,59]. The majority of these foci appear relatively indolent with mitotically inactive neoplastic cells. These cells are small at the neck-zone level and usually enlarge towards the surface of the gastric mucosa exhibiting the distinctive signet ring cell morphology. The proposed histologic model for HDGC development by Carneiro *et al.* starts with signet ring cell carcinoma in situ (Tis), corresponding to the presence of signet ring cells within the basal membrane, and a pagetoid spread pattern of signet ring cells below the preserved epithelium of glands and foveolae within the basal membrane [60]. This is followed by increased pagetoid proliferation of signet ring cells and eventually to invasive carcinoma [60]. Striking is the discrepancy between the numerous T1a carcinomas and most often absence of carcinoma in situ (Tis) lesions, indicating that invasion usually occurs without morphologically detectable carcinoma in situ [60]. Background changes in the gastric mucosa of prophylactic gastrectomy specimens consist of foveolar hyperplasia, tufting of surface epithelium, vacuolization of surface epithelium and mild chronic lymphocytic gastritis without *H.pylori* infection or intestinal metaplasia [41,60,61]. Advanced HDGC presents as a poorly differentiated diffuse carcinoma with sometimes a few signet ring cells (linitis plastica) but also undifferentiated or mixed subtypes with mucinous and sometimes tubular dedifferentiation are seen [Van der Post et al., unpublished data, 2012]. These advanced gastric carcinomas of *CDH1* mutation carriers do not show any characteristics that might discriminate them from sporadic gastric cancers.

### Surveillance endoscopy

The ‘Cambridge surveillance protocol’ is advised for *CDH1* mutation carriers who do not (yet) want to undergo a prophylactic gastrectomy, to individuals at 50% risk of being carrier who are not (yet) willing to be tested for the mutation as well as for members from HDGC families without a known *CDH1* mutation [62]. This protocol comprises *H.Pylori*-testing, annual gastroscopy with ‘high definition’ endoscope, careful inspection of mucosa during 30 minutes, insufflation and desufflation of the stomach, biopsies of mucosal abnormalities and 30 random biopsies from different gastric regions (antrum, angulus, corpus, fundus, cardia) [40]. The endoscopy should be performed using a white light high definition endoscope in a dedicated session with at least 30 minutes allocated to allow for a careful inspection of the mucosa on inflation and deflation, and to allow time for multiple biopsies to be taken [40]. Use of mucolytics such as acetylcysteine may be helpful to obtain good views. Endoscopy permits direct inspection and biopsy of suspicious areas, but diffuse GC is difficult to detect at an early and treatable stage since the lesions tend to spread into the lamina propria without visible exophytic masses. The major problems include difficulties to identify (sub)mucosal lesions and biases in sampling in macroscopically normal-appearing gastric mucosa [63]. Such specimens therefore need to be evaluated by pathologists with expertise with this type of lesions. Several studies have shown that even though *CDH1* mutation carriers had negative biopsies prior to prophylactic gastrectomy, foci were detected in their gastrectomy specimens [49,53,58,59]. Other techniques, such as chromoendoscopic techniques, trimodal imaging, confocal endomicroscopy and molecular imaging techniques are currently not recommended, but need to be further explored in a research setting [40].

## Conclusion

The overall incidence of GC is declining, which is most likely due to the reduction in environmental risk factors. Germline mutations in the *CDH1* gene have been identified as an important cause of HDGC, but still in more than two thirds of strictly selected HDGC families the genetic cause remains unknown. Additionally, the genetic basis of familial cases with an intestinal type gastric cancer is largely unknown. Elucidation of novel gastric cancer susceptibility genes will be an important step towards additional options for gastric cancer prevention. Therefore, identifying new genetic gastric cancer predisposing factors is one of the important targets in the near future.

## Competing interests

The authors declare that they have no competing interests

## Authors’ contributions

All authors contributed to the literature search and manuscript preparation. All authors read and approved the final manuscript.
